# Single-cell transcriptomics deciphers cancer-associated fibroblast heterogeneity and immune regulatory mechanisms in the bladder cancer tumor microenvironment

**DOI:** 10.3389/fcell.2026.1889050

**Published:** 2026-08-28

**Authors:** Yandong He, Wenlong Lu, Guanqun Ju, Lin Chen

**Affiliations:** 1 Department of Urology, Sixth People’s Hospital South Campus Affiliated to Shanghai Jiao Tong University (Shanghai Fengxian District Central Hospital), Shanghai, China; 2 Urology Centre, Shuguang Hospital Affiliated to Shanghai University of Traditional Chinese Medicine, Shanghai, China; 3 Surgical Institute of Integrative Medicine, Shuguang Hospital Affiliated to Shanghai University of Traditional Chinese Medicine, Shanghai, China; 4 Surgical Institute, Shuguang Hospital Affiliated to Shanghai University of Traditional Chinese Medicine, Shanghai, China; 5 Shanghai Key Laboratory of Traditional Chinese Clinical Medicine, Shanghai, China

**Keywords:** bladder cancer, cancer-associated fibroblasts, fibroblast heterogeneity, single-cell RNA sequencing, tumor microenvironment

## Abstract

**Background:**

Bladder cancer (BLCA) is a common malignancy, with muscle-invasive bladder cancer (MIBC) associated with a 5-year survival rate below 50%. Cancer-associated fibroblasts (CAFs) are heterogeneous stromal components of the tumor microenvironment (TME) that contribute to tumor progression, therapy resistance, and immune evasion. However, the molecular basis of CAF diversity and immune regulation in BLCA remains incompletely understood.

**Methods:**

We analyzed single-cell transcriptomic data from GEO dataset GSE135337 using dimensionality reduction, unsupervised clustering, differential expression analysis, GO/KEGG/GSEA, diffusion pseudotime inference, and LIANA ligand–receptor analysis. TCGA-BLCA bulk transcriptomic data (n = 408 total; n = 401 after stage filtering) were used for score validation. RT–qPCR was performed to assess selected myCAF- and iCAF-associated genes in RT4 and T24 bladder cancer cells.

**Results:**

Analysis of 9,253 post-QC cells across two non-paired specimens (6,035 adjacent-tissue; 3,218 tumor) yielded 9 DE-marker-annotated cell populations. Among 1,923 retained adjacent-tissue fibroblasts, two transcriptionally distinct states were identified: Homeostatic fibroblasts (n = 1,197; enriched for COL1A1, DCN) and CCL2-high activated-like fibroblasts (n = 726; CCL2 log2FC = 0.806, FDR = 8.08 × 10^−11^). No fibroblast-like cells were retained in the tumor specimen. Diffusion pseudotime positioned CCL2-high cells at a later within-sample state (median DPT 0.813 vs. 0.672; FBLN1 rho = −0.808). Exploratory LIANA analysis identified 1,347 fibroblast-outgoing ligand–receptor interactions. A pre-specified six-gene myCAF score (ACTA2, COL1A1, MMP11, MYL9, TAGLN, TPM2) tracked pathological stage in TCGA-BLCA (P = 1.60 × 10^−12^) and showed an unadjusted overall survival association (HR = 1.21, 95% CI: 1.02–1.42, P = 0.025) that attenuated to non-significance after age and stage adjustment (HR = 1.03, P = 0.748); the association was not replicated in GSE31684 (n = 93; log-rank P = 0.443). RT–qPCR confirmed higher expression of ACTA2, POSTN, MMP11, FAP, IL6, and CXCL12 in T24 versus RT4 cells (4.33–16.56-fold; all BH-adjusted q < 0.01).

**Conclusion:**

Single-cell transcriptomics of publicly available BLCA data identifies two transcriptionally distinct fibroblast states in adjacent tissue and supports an exploratory ligand–receptor interaction framework. A six-gene myCAF-associated score tracks pathological stage but does not independently predict overall survival after covariate adjustment and was not replicated in an independent cohort. These findings constitute an exploratory computational framework warranting prospective validation with primary CAF populations and adequately powered multi-specimen cohorts.

## Introduction

Bladder cancer (BLCA) is one of the most common malignancies of the urinary tract, accounting for approximately 573,000 new cases and 212,000 deaths globally in 2020 (Sung et al., 2021). Non-muscle-invasive bladder cancer (NMIBC) has a favorable prognosis with transurethral resection and intravesical immunotherapy, but exhibits high recurrence rates exceeding 70% at 5 years (Babjuk et al., 2022). Muscle-invasive bladder cancer (MIBC), representing approximately 25% of all BLCA cases, is characterized by aggressive local invasion and frequent metastasis, with a 5-year overall survival of less than 50% even after radical cystectomy with cisplatin-based chemotherapy (Alfred et al., 2017). Immune checkpoint inhibitors (ICIs), particularly anti-PD-L1 agents including atezolizumab and durvalumab, have yielded objective response rates of only 15%–25% in metastatic BLCA, underscoring the critical need to understand mechanisms of primary and acquired resistance (Powles et al., 2020; Rosenberg et al., 2016).

The tumor microenvironment (TME) plays a central role in dictating BLCA progression, immune evasion, and therapeutic responsiveness. Among TME components, cancer-associated fibroblasts (CAFs) represent a major and functionally diverse stromal population that modulates extracellular matrix (ECM) composition, cytokine networks, angiogenesis, and immune cell trafficking (Kalluri, 2016). Dysregulation of cytokine signalling networks—including IL-6 and related inflammatory pathways—has emerged as a common pathophysiological mechanism across both oncological and non-oncological inflammatory contexts, reflecting the fundamental role of cytokines in stromal–immune crosstalk (Li et al., 2025). CAFs are not a single cell type but rather a heterogeneous collection of functionally distinct populations with divergent and sometimes opposing roles in tumor biology. Seminal work by Öhlund et al. (Öhlund et al., 2017) and Costa et al. (Costa et al., 2018) established the existence of functionally distinct CAF subtypes in pancreatic and breast cancer, respectively, characterizing inflammatory CAFs (iCAFs) enriched for cytokine secretion and myofibroblastic CAFs (myCAFs) enriched for ECM-contractile gene programs. Subsequent studies have identified additional CAF subtypes including antigen-presenting CAFs (apCAFs) capable of MHC II-mediated T cell regulation (Elyada et al., 2019).

In BLCA specifically, stromal fibroblast activation has been associated with reduced immune infiltration, TGF-β-mediated immune exclusion, and poor response to immunotherapy—as demonstrated in the landmark IMvigor210 trial analysis by Mariathasan et al. (Mariathasan et al., 2018). However, the precise identity, heterogeneity, and specific immunomodulatory functions of distinct CAF populations in the BLCA TME remain incompletely defined. Bulk transcriptomic approaches have identified CAF-related gene signatures associated with clinical outcomes (Robertson et al., 2017), but lack the cellular resolution to distinguish individual CAF subtypes and their distinct contributions.

The advent of single-cell RNA sequencing (scRNA-seq) has transformed our ability to resolve TME heterogeneity at cellular resolution (Cao et al., 2019). Recent scRNA-seq studies of BLCA have characterized the global cellular landscape of the BLCA TME (Chen et al., 2020; Zheng et al., 2017), but the molecular definition of CAF subtypes, their developmental trajectories, transcriptional regulatory networks, and immune-regulatory mechanisms have not been comprehensively characterized. The GEO repository contains several high-quality BLCA scRNA-seq datasets including GSE135337, GSE173822, and GSE240429 that collectively encompass diverse disease stages and treatment contexts.

In the present study, we performed an exploratory re-analysis of publicly available BLCA single-cell transcriptomic data (GSE135337) with focus on fibroblast-state characterisation. We identified two transcriptionally distinct adjacent-tissue fibroblast states, characterised their within-sample state continuum using diffusion pseudotime, and explored ligand–receptor interaction patterns using LIANA. A pre-specified six-gene myCAF-associated score was evaluated in three bulk cohorts (TCGA-BLCA, GSE31684, GSE13507). Selected CAF-marker genes were additionally assessed by RT–qPCR in bladder cancer cell lines of contrasting invasive phenotypes. Our work provides an exploratory molecular characterisation of BLCA fibroblast states within the scope and limitations of the available public dataset.

## Methods

### Public datasets and analytic design

This real-data re-analysis used public bladder-cancer datasets supplied with the project (Barrett et al., 2013). Single-cell expression matrices were from GSE135337: GSM4006644 (BC1 tumor specimen) and GSM5329919 (BCN/BC4 paracancerous specimen). The two single-cell specimens are non-paired and provide one biological specimen per condition. Bulk analyses used TCGA-BLCA, GSE31684 (GPL570), and GSE13507 (GPL6102).

### Single-cell matrix processing and quality control

GEO-provided integer gene-by-cell matrices were read after gene-symbol de-duplication. Because the GEO supplementary description reports median-scaled expression, the supplied scale was retained and log1p transformed without an additional library-size normalization. Cells with fewer than 200 detected genes, a matrix total below 500, or mitochondrial signal above 25% were excluded. Quality-control displays are therefore labeled matrix total and detected genes rather than unverified molecule-count labels.

### Embedding, DE-based annotation, and fibroblast refinement

Highly variable genes were selected after log transformation, followed by scaling, PCA, nearest-neighbor graph construction, UMAP, and Leiden clustering using a fixed seed (Hao et al., 2021; Wolf et al., 2018; McInnes et al., 2018; Traag et al., 2019). Clusters were annotated from cluster-versus-rest differential-expression markers and marker-set overlap, rather than by per-cell signature assignment. Broad fibroblast-labelled cells were re-clustered; immune-like and epithelial-like contaminant clusters were excluded before retained fibroblast-state analysis.

### Fibroblast-state, DPT, and interaction analyses

Retained fibroblast states were summarized with log1p expression and cluster-level differential-expression statistics. Diffusion pseudotime was rooted in the retained adjacent-tissue fibroblast state with high extracellular-matrix and low activation scores (Subramanian et al., 2005). LIANA consensus ligand-receptor ranking used 250 permutations after removal of broad-fibroblast contaminants. These analyses are exploratory because the local single-cell data contain one biological specimen per condition and no spatial validation (Yu et al., 2012; Dimitrov et al., 2022).

### TCGA-BLCA bulk score and survival analysis

TCGA raw counts were filtered to retain genes with at least 10 counts in at least 10% of samples, converted to counts per million, and log2 transformed. One deterministic primary-tumor aliquot per patient was retained for patient-level survival analysis (Love et al., 2014). A fixed six-gene myCAF-associated score (ACTA2, COL1A1, MMP11, MYL9, TAGLN, and TPM2), defined before bulk scoring and not selected from the local single-cell clustering result, was calculated as the mean gene-wise z-score. Overall survival was assessed using a pre-specified median-split Kaplan-Meier/log-rank analysis (cutoff = median score value, −0.028; no data-driven cutpoint optimization was applied), univariable Cox regression, and a penalized age-stage-adjusted Cox model after exclusion of unknown-stage cases.

### External GEO bulk analyses and statistics

GSE31684 and GSE13507 matrices were mapped to gene symbols using their supplied GPL annotations before six-gene score calculation. GSE13507 source categories were reconstructed from GEO biological-source metadata: healthy normal mucosa, histologically normal mucosa surrounding cancer, primary tumor, and recurrent non-muscle-invasive tumor. Two-sided Mann-Whitney, Kruskal–Wallis, and Spearman tests were used as displayed; all reported values were calculated from the stored analysis objects and tables.

### Cell culture

Human bladder cancer cell lines RT4 and T24 were obtained from the American Type Culture Collection (ATCC, Manassas, VA, USA). RT4 was used as a low-invasive bladder cancer cell model, and T24 was used as a high-invasive model. These cell lines were selected to examine whether bladder cancer cells with contrasting invasive phenotypes differ in expression of genes associated with the major CAF programs identified bioinformatically, providing preliminary cell-autonomous evidence for these molecular signatures. Cells were maintained in RPMI-1640 medium containing 10% fetal bovine serum and 1% penicillin/streptomycin at 37 °C in a humidified incubator with 5% CO_2_. Cells in the logarithmic growth phase were collected for RNA extraction.

### RNA extraction and RT-qPCR

Total RNA was isolated from RT4 and T24 cells using TRIzol reagent following the manufacturer’s instructions. RNA quantity and purity were measured with a NanoDrop spectrophotometer. cDNA was synthesized using a reverse transcription kit, and quantitative PCR was carried out with SYBR Green qPCR Master Mix on a real-time PCR system. The mRNA levels of ACTA2, POSTN, MMP11, FAP, IL6, and CXCL12 were normalized to GAPDH. Relative expression was calculated using the 2^-ΔΔCt method, with RT4 cells set as the reference group. Each experiment was performed with three biological replicates. Primer sequences are provided in Supplementary Table S1.

### Statistical analysis

All analyses were performed in R v4.3.0 and Python 3.10. Two-sided statistical tests were used throughout. Multiple testing correction applied the Benjamini–Hochberg FDR method. Survival curves were compared by log-rank test. Hazard ratios with 95% confidence intervals are reported. Spearman or Pearson correlation coefficients with p-values are reported. Statistical significance threshold: p < 0.05 (or FDR<0.05 for multiple comparisons). *In vitro* RT-qPCR data are presented as the mean ± standard deviation (SD) from three independent experiments. Differences between groups were assessed using one-way analysis of variance (ANOVA) followed by Tukey’s *post hoc* test in GraphPad Prism 9.0. A two-sided p-value <0.05 was considered statistically significant.

## Results

### Single-cell profiling resolved an atlas across the adjacent and tumor specimens

**FIGURE 1:**
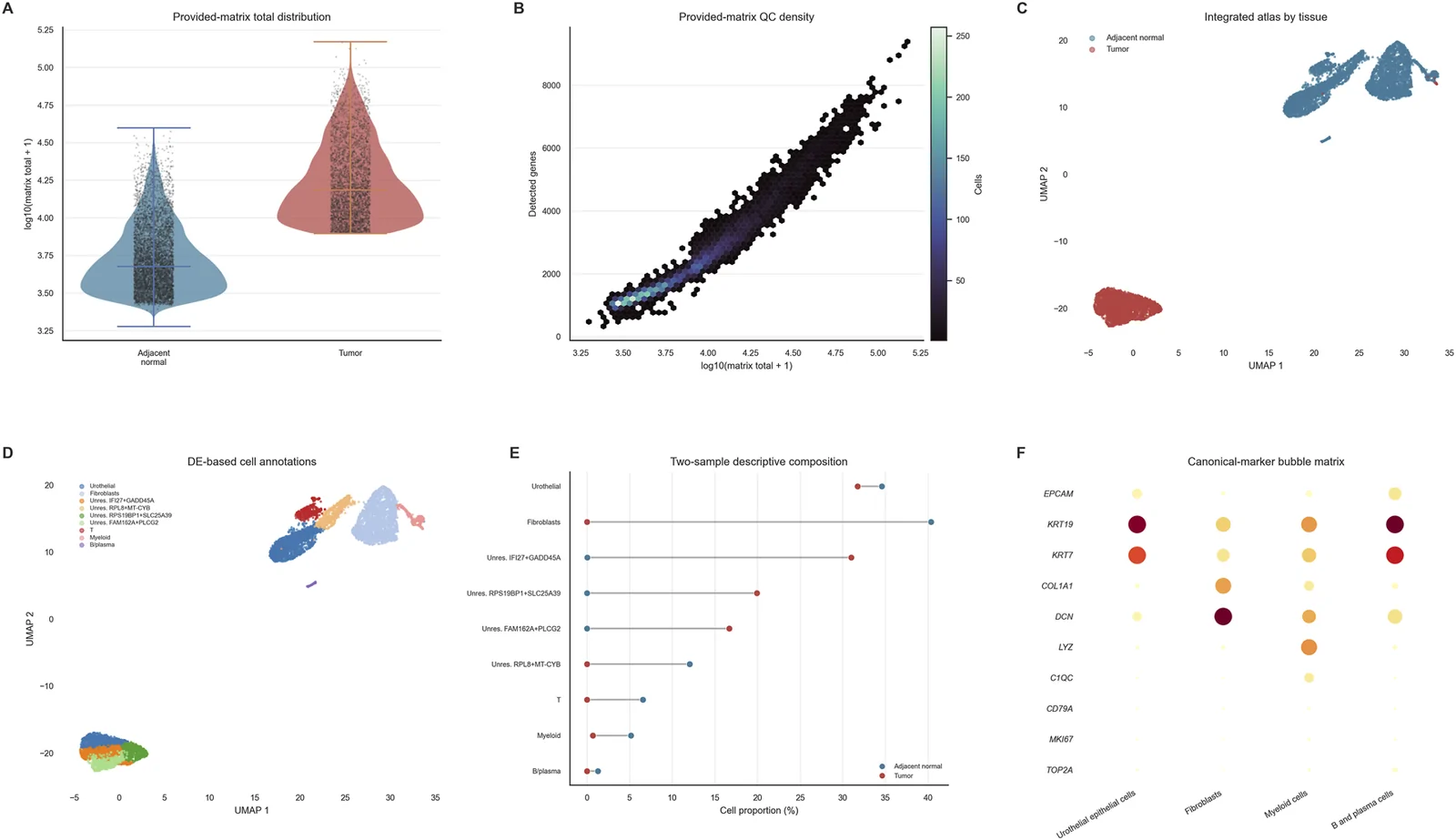
GEO-provided single-cell matrix QC and DE-based atlas annotation. **(A)** provided-matrix total distribution by specimen. **(B)** Matrix-total versus detected-gene density. **(C)** Iintegrated UMAP by specimen. **(D)** DE-marker-derived biological annotations. **(E)** Descriptive composition across the two non-paired specimens. **(F)** Canonical marker bubble matrix.

The provided-matrix total distribution included 6,035 adjacent-tissue cells and 3,218 tumor-specimen cells; median matrix totals were 4759.0 and 15,338.5, respectively (Figure 1A). Median detected-gene counts were 1486.0 in adjacent tissue and 3481.0 in the tumor specimen; the panel uses the supplied matrix, not raw-count labels (Figure 1B). The integrated UMAP displays 9,253 post-QC cells across the two non-paired specimens (Figure 1C). DE-marker annotation yielded nine biological labels; the largest labels were Urothelial epithelial cells (n = 3,109) and Fibroblasts (n = 2,435) (Figure 1D). This two-sample composition view is descriptive: fibroblast-like cells numbered 2,435 in adjacent tissue and 0 in the tumor specimen (Figure 1E). Mean log1p expression of COL1A1 and DCN in the broad fibroblast label was 1.419 and 3.614, respectively, supporting the marker matrix display (Figure 1F). *GSM4006644 and GSM5329919 are one non-paired biological specimen per condition; this figure does not support patient-level differential inference.*

### The tumor specimen contained heterogeneous DE-marker-defined cell states

**FIGURE 2:**
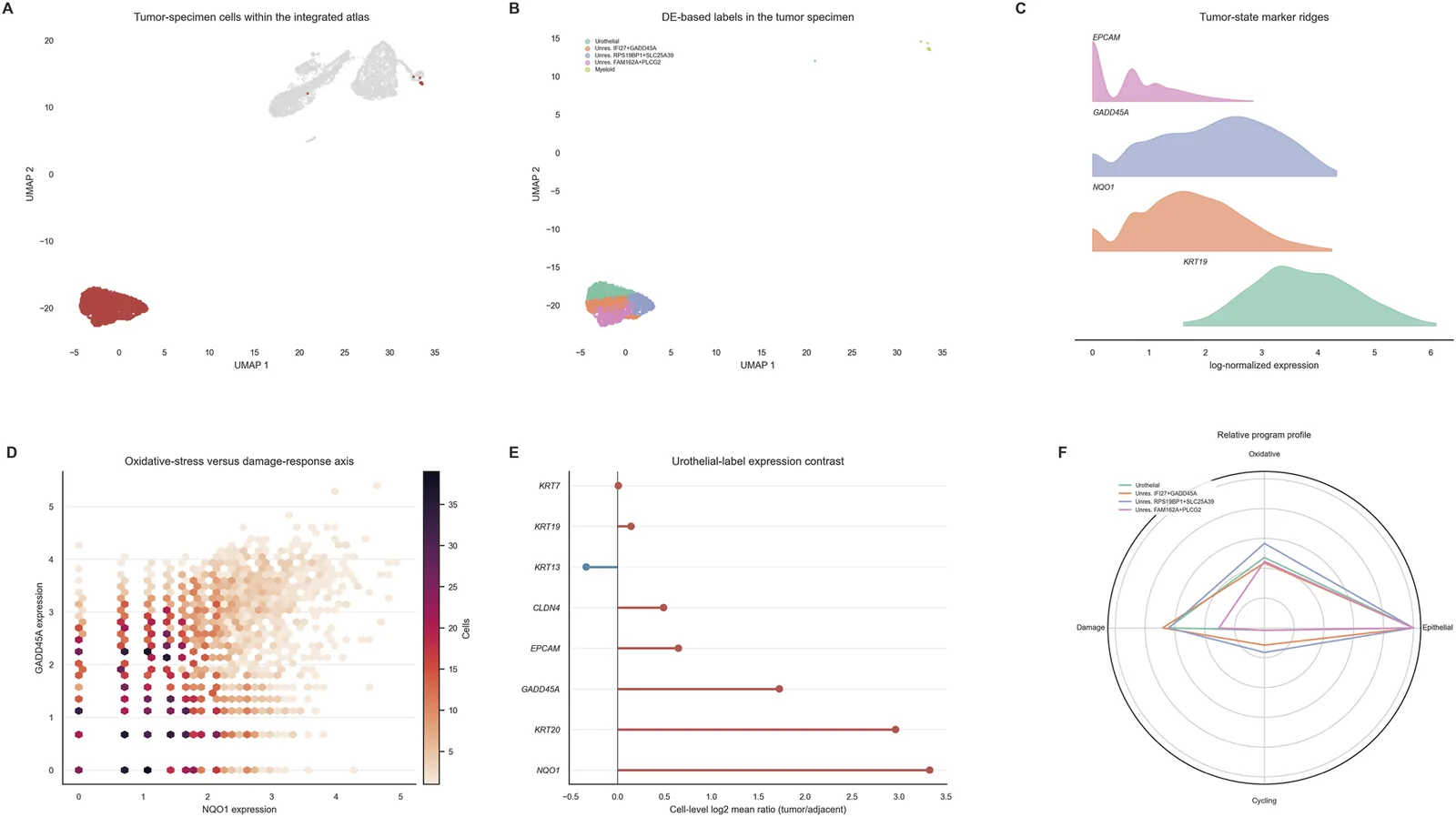
Descriptive DE-based cell labels in the tumor specimen. **(A)** Cells from the tumor specimen highlighted in the integrated atlas. **(B)** Persisted DE-marker labels without plot-layer relabeling. **(C)** Marker-expression ridges. **(D)** NQO1-GADD45A cell-level expression density. **(E)** Exploratory tumor-versus-adjacent cells carrying the urothelial label mean-ratio display. **(F)** Relative gene-program profile for the most abundant tumor labels.

The tumor-specimen overlay contains 3,218 cells from GSM4006644; these are specimen-derived cells and were not inferred to be malignant (Figure 2A). DE-marker annotation of the tumor specimen yielded five labels; the two most abundant were Urothelial epithelial cells (n = 1,021) and Unresolved IFI27+GADD45A (n = 997). No figure-specific label remapping was applied (Figure 2B). Tumor-specimen median log1p expression was 3.714 for KRT19 and 1.792 for NQO1 (Figure 2C). NQO1 and GADD45A showed a cell-level Spearman rho of 0.269 (P = 1.36e-54) within the tumor specimen (Figure 2D). The largest displayed exploratory cell-level log2 mean ratio was NQO1 = 3.326 (tumor versus adjacent cells carrying the urothelial label) (Figure 2E). The radial profile summarizes four gene programs across the four most abundant tumor DE-marker labels and is descriptive rather than an independent validation (Figure 2F). *Tumor versus adjacent contrasts are cell-level exploratory comparisons between one non-paired specimen per condition.*

### Adjacent tissue contained homeostatic and CCL2-high activated-like fibroblast states

**FIGURE 3:**
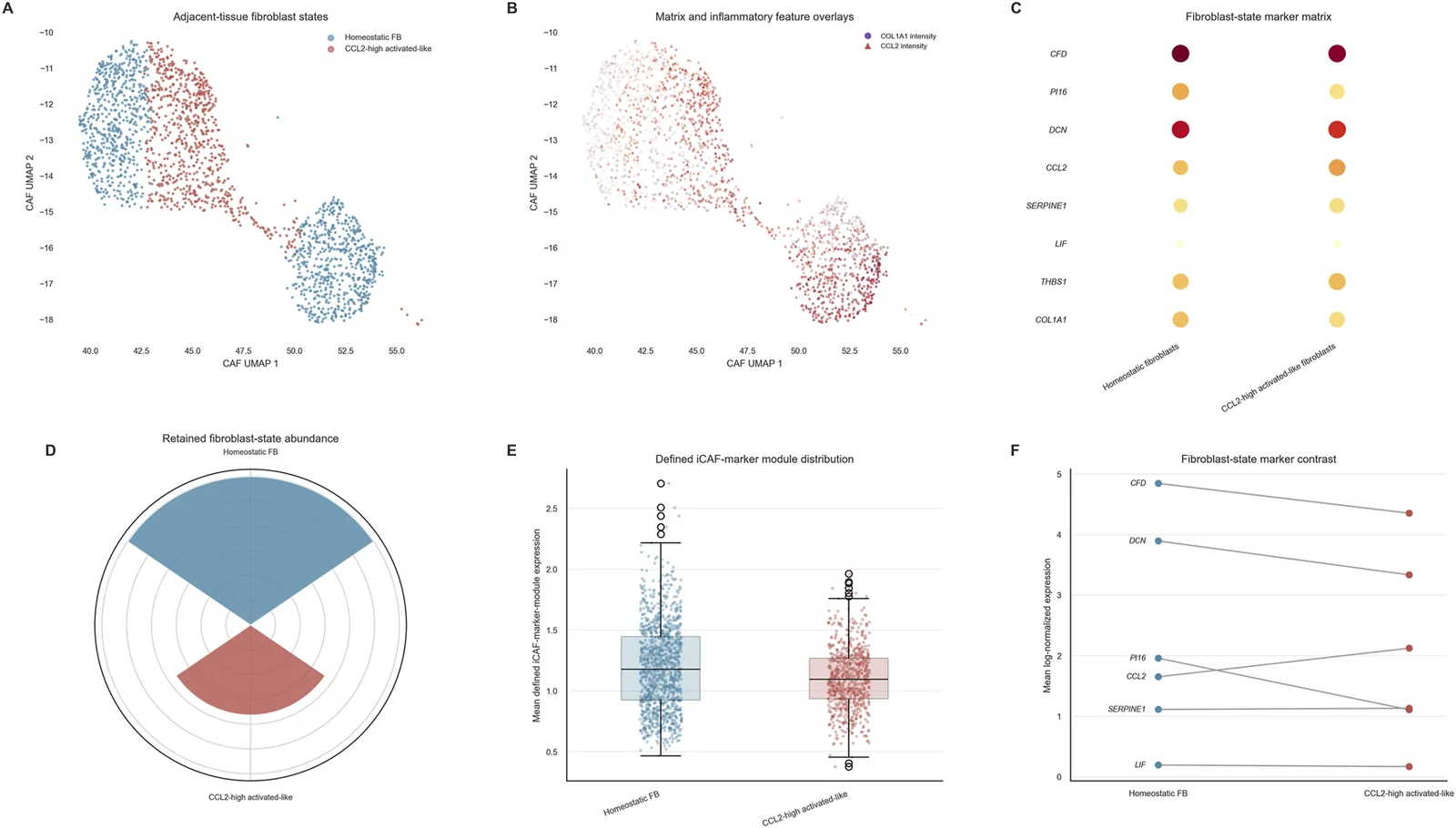
Retained adjacent-tissue fibroblast states after contaminant exclusion. **(A)** Fibroblast UMAP. **(B)** COL1A1 and CCL2 feature overlays. **(C)** Marker bubble matrix. **(D)** State abundance. **(E)** Defined iCAF-marker-module score distribution, not used for state-label validation. **(F)** Fibroblast-state marker contrast.

The retained fibroblast object contains 1,923 adjacent-tissue fibroblasts: Homeostatic fibroblasts (n = 1,197); CCL2-high activated-like fibroblasts (n = 726) (Figure 3A). Across retained fibroblasts, mean log1p COL1A1 expression was 1.481 and mean CCL2 expression was 1.828 (Figure 3B). CCL2-high activated-like-state CCL2 log2FC was 0.806 with FDR 8.08e-11; homeostatic CFD log2FC was 0.717 with FDR 1.49e-89 (Figure 3C). The polar abundance display represents 1,923 retained cells and no retained local myCAF-like state (Figure 3D). Median defined iCAF-marker-module scores were 1.178 for homeostatic fibroblasts and 1.096 for CCL2-high activated-like fibroblasts (Mann-Whitney P = 2.28e-08); this broad module did not define the CCL2-high label (Figure 3E). Mean CCL2 expression was 1.650 versus 2.123 across the two retained states (Figure 3F). *All retained fibroblasts are from the adjacent-tissue specimen; cell-level P values quantify within-specimen state separation, not patient-level or population-level inference.*

### DPT placed CCL2-high activated-like fibroblasts toward the late end of a within-sample state continuum

**FIGURE 4:**
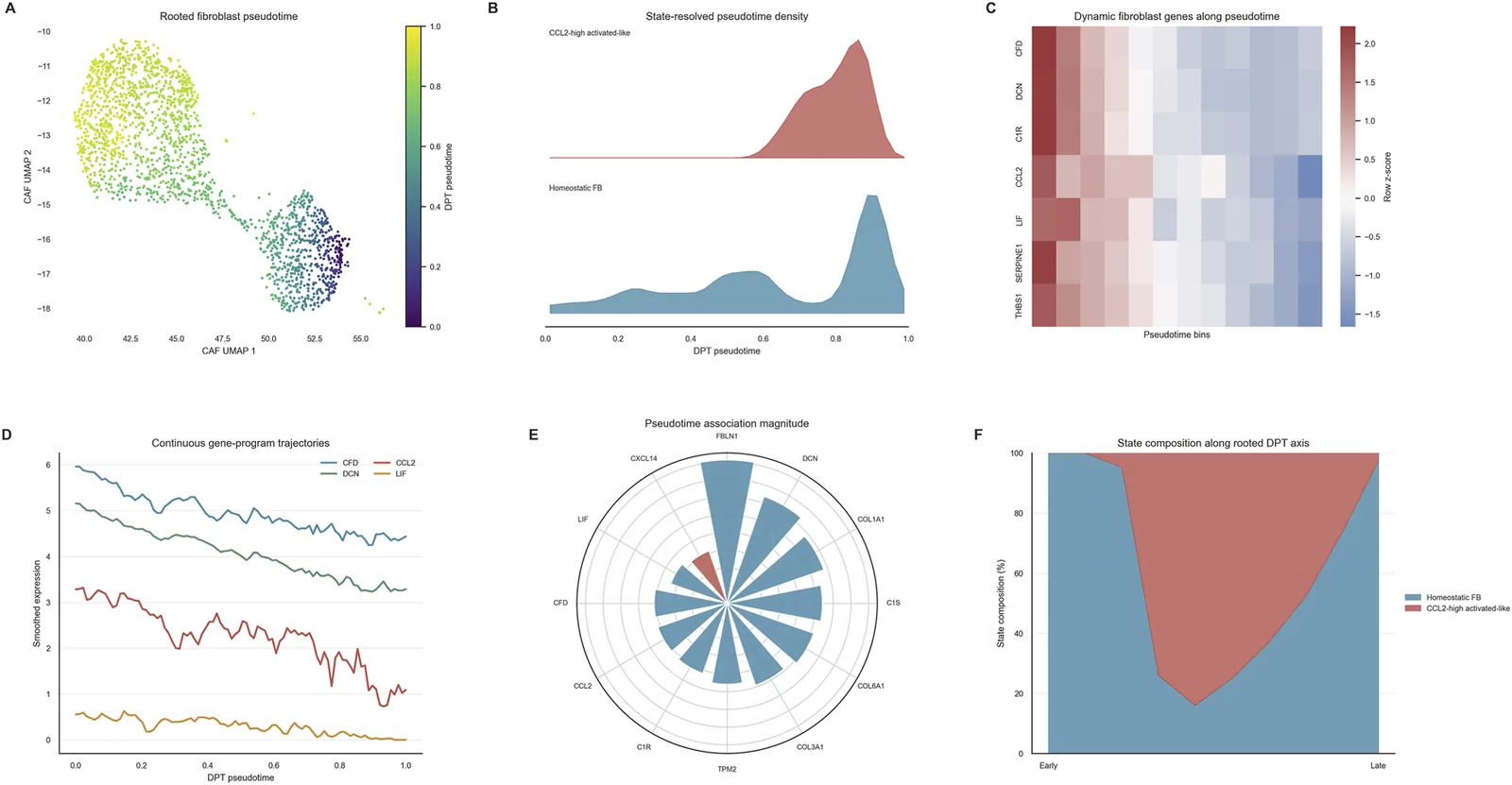
Rooted within-sample DPT ordering of retained adjacent-tissue fibroblasts. **(A)** Rooted DPT UMAP. **(B)** State-resolved DPT densities. **(C)** Dynamic-gene heatmap. **(D)** Continuous gene profiles over DPT. **(E)** Absolute pseudotime-association magnitudes. **(F)** State composition along the rooted DPT coordinate.

DPT was calculated for 1,923 retained fibroblasts with a pseudotime range of 0.000–1.000 (Figure 4A). Median DPT pseudotime was 0.672 for homeostatic fibroblasts and 0.813 for CCL2-high activated-like fibroblasts (Figure 4B). The top dynamic marker by absolute correlation was FBLN1 (rho = −0.808, P < 2.23e-308) (Figure 4C). Continuous profiles plot the same rooted DPT coordinate and do not establish a temporal lineage (Figure 4D). The polar chart summarizes 12 genes; the strongest absolute rho was 0.808 (Figure 4E). CCL2-high activated-like-state composition was 0.0% in the earliest decile and 2.6% in the latest decile (Figure 4F). *Rooted DPT is a within-sample state ordering, not evidence of fibroblast conversion, lineage, or disease progression; its cell-level P values are not patient-level inference.*

### Exploratory ligand-receptor analysis highlighted fibroblast-linked interaction patterns

**FIGURE 5:**
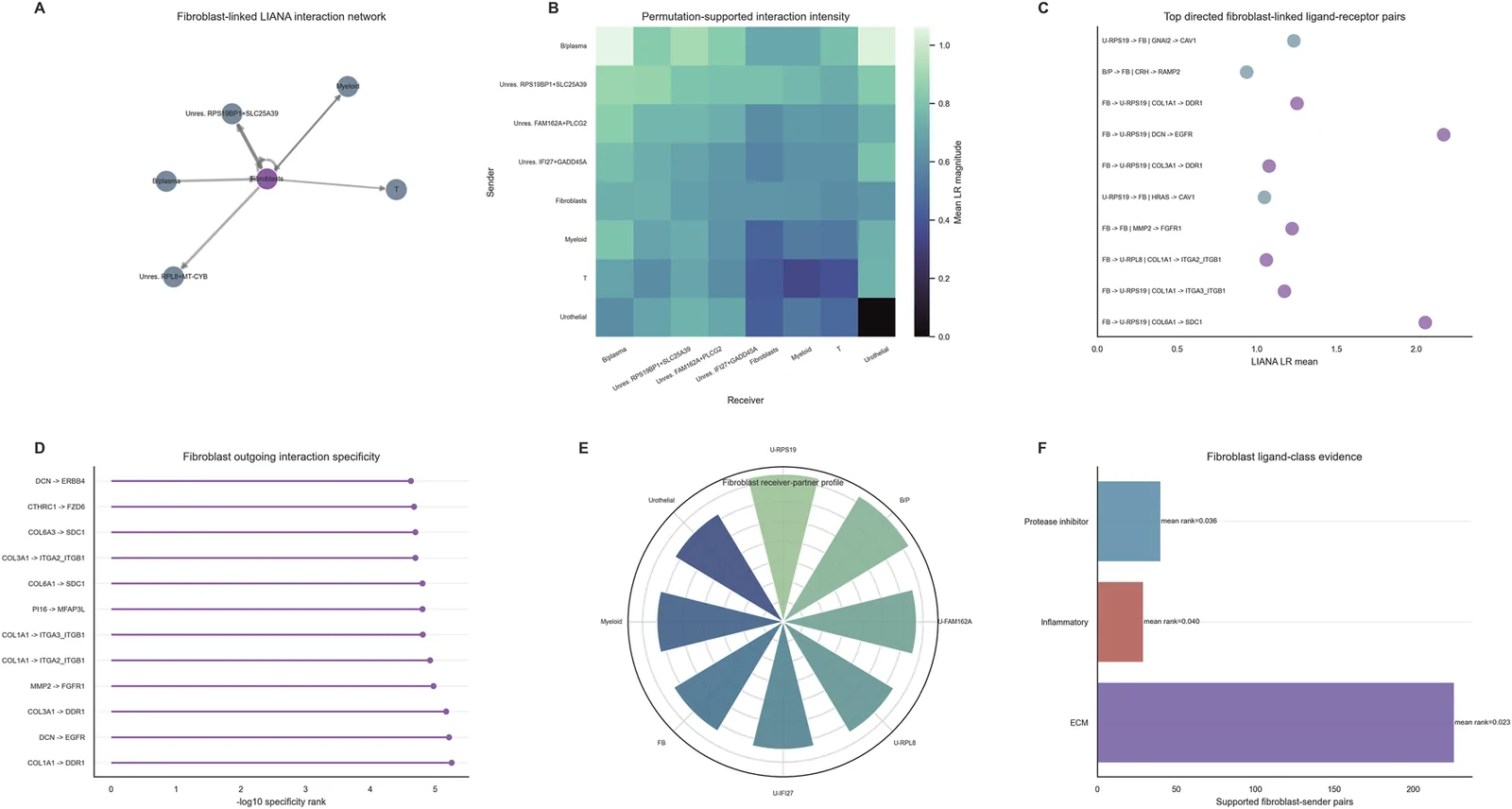
Exploratory fibroblast-linked LIANA interaction ranking after contaminant exclusion. **(A)**, Top directed cell-type network. **(B)** Permutation-supported interaction intensity. **(C)** Top directed fibroblast-linked ligand-receptor pairs with explicit sender and receiver labels. **(D)** Fibroblast outgoing specificity. **(E)** Fibroblast receiver-partner profile. **(F)** Fibroblast ligand-class evidence.

LIANA used 8,741 cells after excluding fibroblast-cluster contaminants; the plotted network contains 16 top directed cell-type edges (Figure 5A). The permutation-supported heatmap includes nine senders and nine receivers under the displayed evidence filter (Figure 5B). The top fibroblast-linked pair had sender Unresolved RPS19BP1+SLC25A39 and receiver Fibroblasts: GNAI2 - > CAV1 (LR mean = 1.232, specificity rank = 0.0000) (Figure 5C). Fibroblast-sender interactions passing the displayed evidence filter numbered 1,347 (Figure 5D). The receiver-partner profile includes nine fibroblast target categories among supported outgoing interactions (Figure 5E). Fibroblast-sender evidence contained 226 ECM, 29 inflammatory, and 40 protease-inhibitor pairs (Figure 5F). *LIANA used 250 permutations and remains exploratory; it is not spatial validation and does not establish clinical causality.*

### The six-gene CAF score tracked stage and showed an unadjusted, but not adjusted, survival association in TCGA-BLCA

**FIGURE 6:**
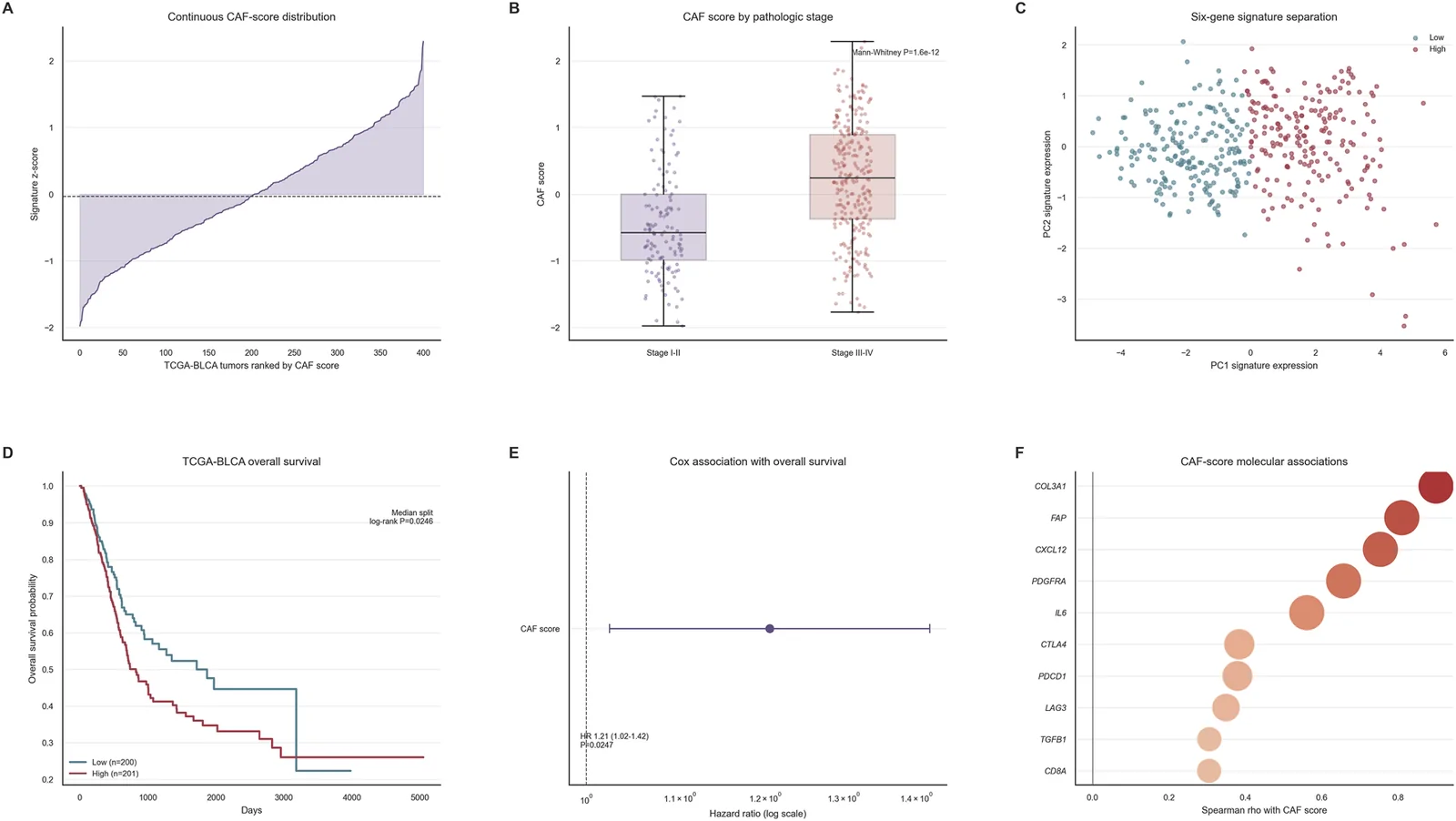
TCGA-BLCA six-gene CAF-score associations. **(A)** Continuous score distribution. **(B)** Score by pathologic stage. **(C)** PCA of the six score genes. **(D)** Median-split overall survival. **(E)** Univariable Cox association. **(F)** Molecular correlations with the score.

The TCGA-BLCA score distribution included n = 401 tumors, with median score −0.028 and median-split cutoff −0.028 (Figure 6A). Stage I-II (n = 129) and stage III-IV (n = 270) scores differed by Mann-Whitney P = 1.60e-12 (Figure 6B). PCA uses the same six genes used to construct the score and visualizes score construction rather than an independent molecular validation (Figure 6C). Median-split overall-survival log-rank P = 0.025 with 176 events (Figure 6D). Univariable Cox HR was 1.21 (95% CI 1.02–1.42; P = 0.025); age-stage-adjusted HR was 1.03 (P = 0.748) (Figure 6E). The strongest displayed molecular association was COL3A1 (rho = 0.899, P = 6.44e-145) (Figure 6F). *The univariable association attenuated after age and stage adjustment and should not be described as an independent prognostic effect.*

### GSE31684 did not validate a survival association for the six-gene CAF score

**FIGURE 7:**
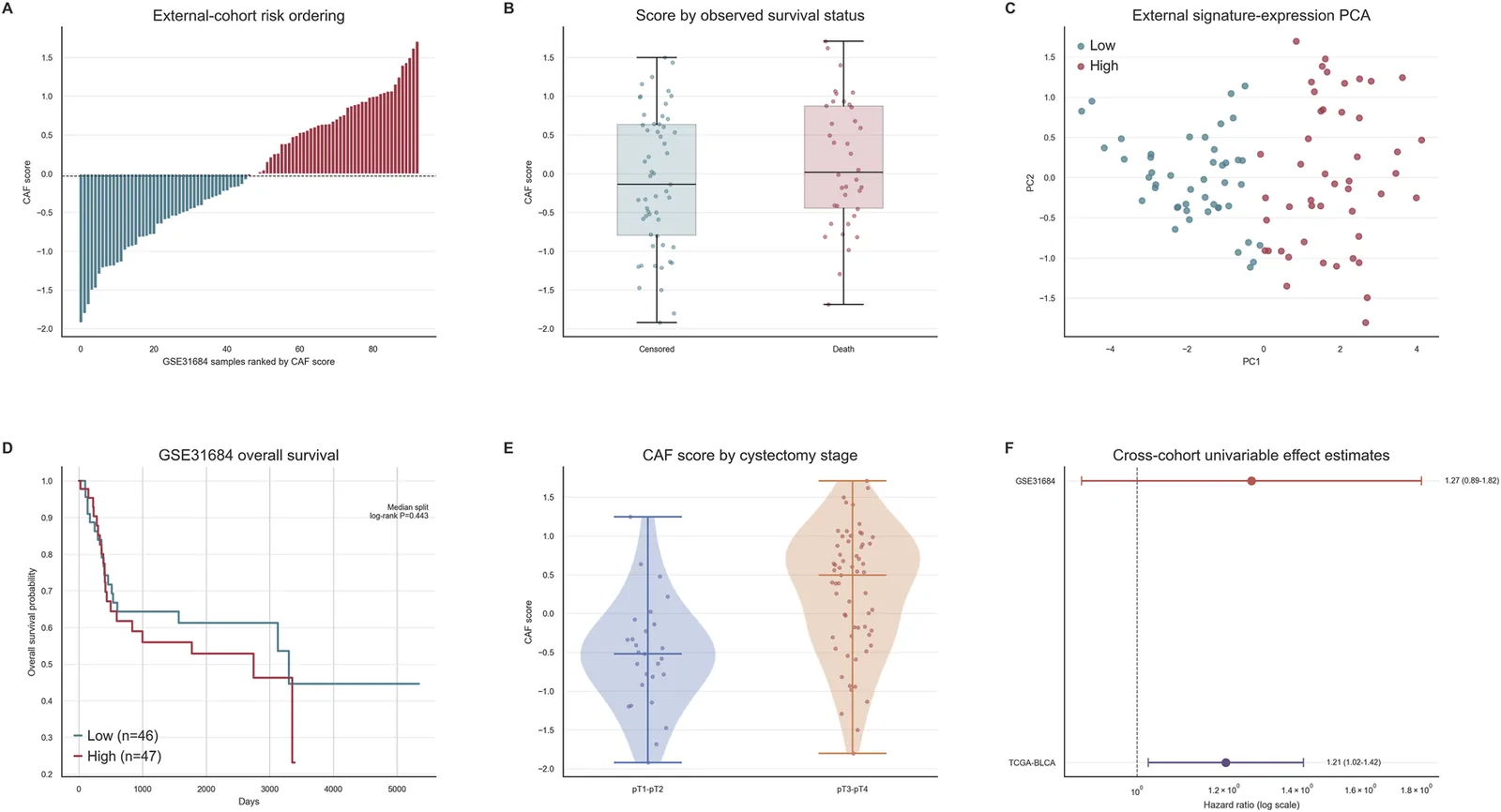
External GSE31684 assessment of the six-gene CAF score. **(A)** Ranked external score distribution. **(B)** Score by observed survival status. **(C)** PCA of the six score genes. **(D)** Median-split overall survival. **(E)** Score by cystectomy stage. **(F)** Cross-cohort univariable Cox estimates.

The external cohort included n = 93 samples with median score −0.027 (Figure 7A). CAF score medians were −0.137 for censored samples (n = 55) and 0.019 for deaths (n = 38; Mann-Whitney P = 0.224) (Figure 7B). PCA uses the six score components and therefore visualizes the score representation rather than an independent validation (Figure 7C). Median-split survival log-rank P = 0.443 with 38 events (Figure 7D). Cystectomy stage groups contained 27 pT1-pT2 and 61 pT3-pT4 samples (Figure 7E). Univariable HR was 1.27 (95% CI 0.89–1.82; P = 0.186); adjusted HR was 0.91 (P = 0.625) (Figure 7F). *Neither univariable nor adjusted GSE31684 survival association was statistically significant.*

### GSE13507 score patterns differed across source-defined tissue groups and invasiveness

**FIGURE 8:**
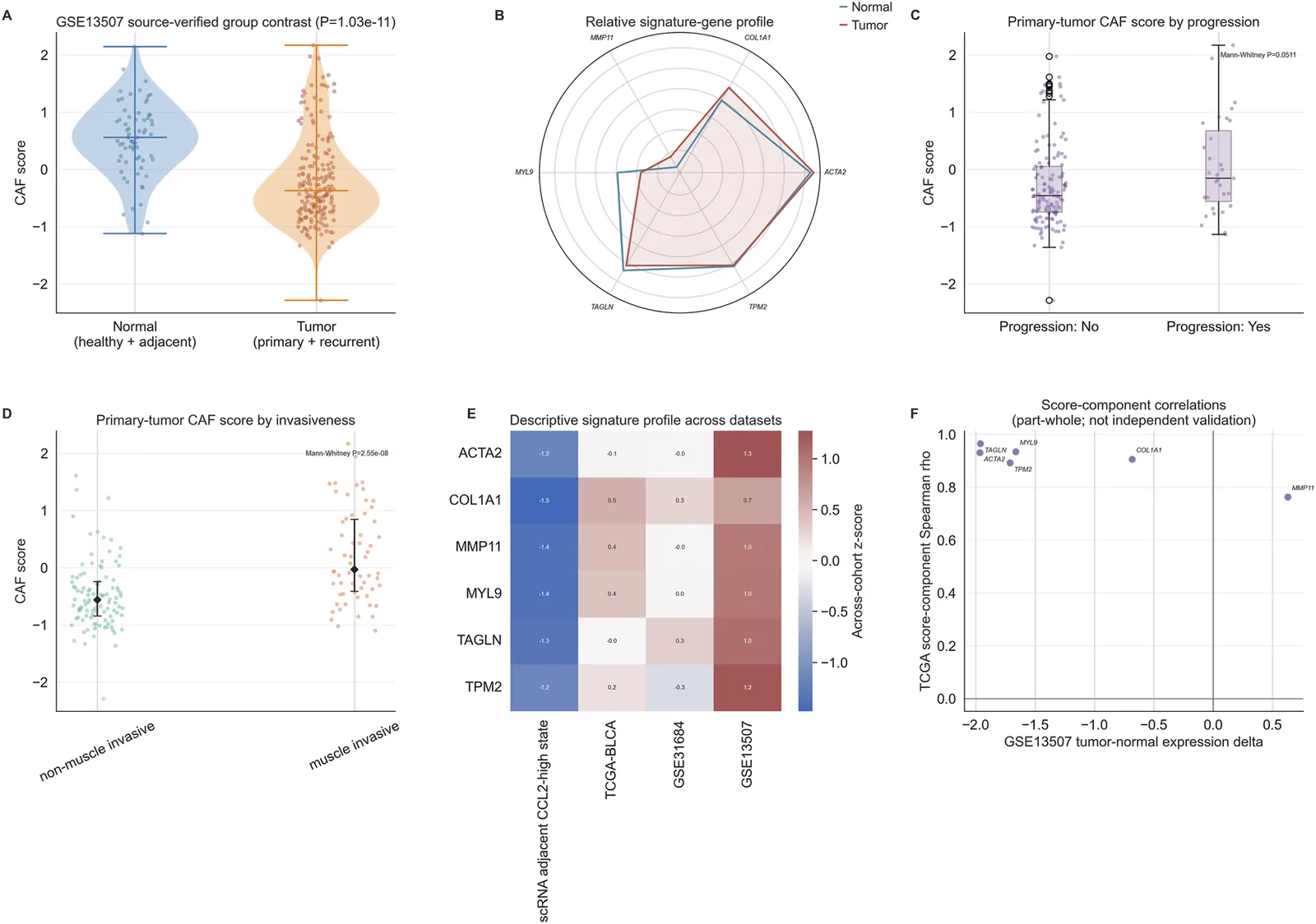
Source-verified GSE13507 score groups and descriptive cross-cohort profiles. **(A)** Combined normal versus tumor score contrast reconstructed from GEO source metadata. **(B)** Relative six-gene profile. **(C)** Primary-tumor score by progression. **(D)** Primary-tumor score by invasiveness. **(E)** Descriptive cross-dataset row-z profile. **(F)** GSE13507 expression deltas against TCGA score-component correlations; this is a part-whole display, not independent validation.

Source-verified normal samples (n = 67; healthy n = 9, adjacent n = 58) and tumors (n = 188; primary n = 165, recurrent n = 23) had mean scores 0.531 and −0.195; Kruskal P = 1.03e-11 (Figure 8A). The radial plot compares relative six-gene profiles between the source-verified combined normal and tumor groups (Figure 8B). Among primary tumors, progression No (n = 134) versus Yes (n = 31) had Mann-Whitney P = 0.051 (Figure 8C). Primary-tumor invasiveness groups non-muscle invasive (n = 103); muscle invasive (n = 62) had Mann-Whitney P = 2.55e-08 (Figure 8D). Cross-dataset row z-scores are descriptive because they combine distinct single-cell and microarray measurement scales (Figure 8E). The strongest TCGA score-component correlation was TAGLN (rho = 0.966, P = 2.63e-236); because the gene is a score component, this part-whole result is not an independent association, and its GSE13507 tumor-normal expression delta was −1.961 (Figure 8F). *GSE13507 group labels were reconstructed from GEO biological-source metadata; the combined normal group includes histologically normal surrounding mucosa.*

### Expression of CAF-Associated genes in RT4 and T24 cells

**FIGURE 9:**
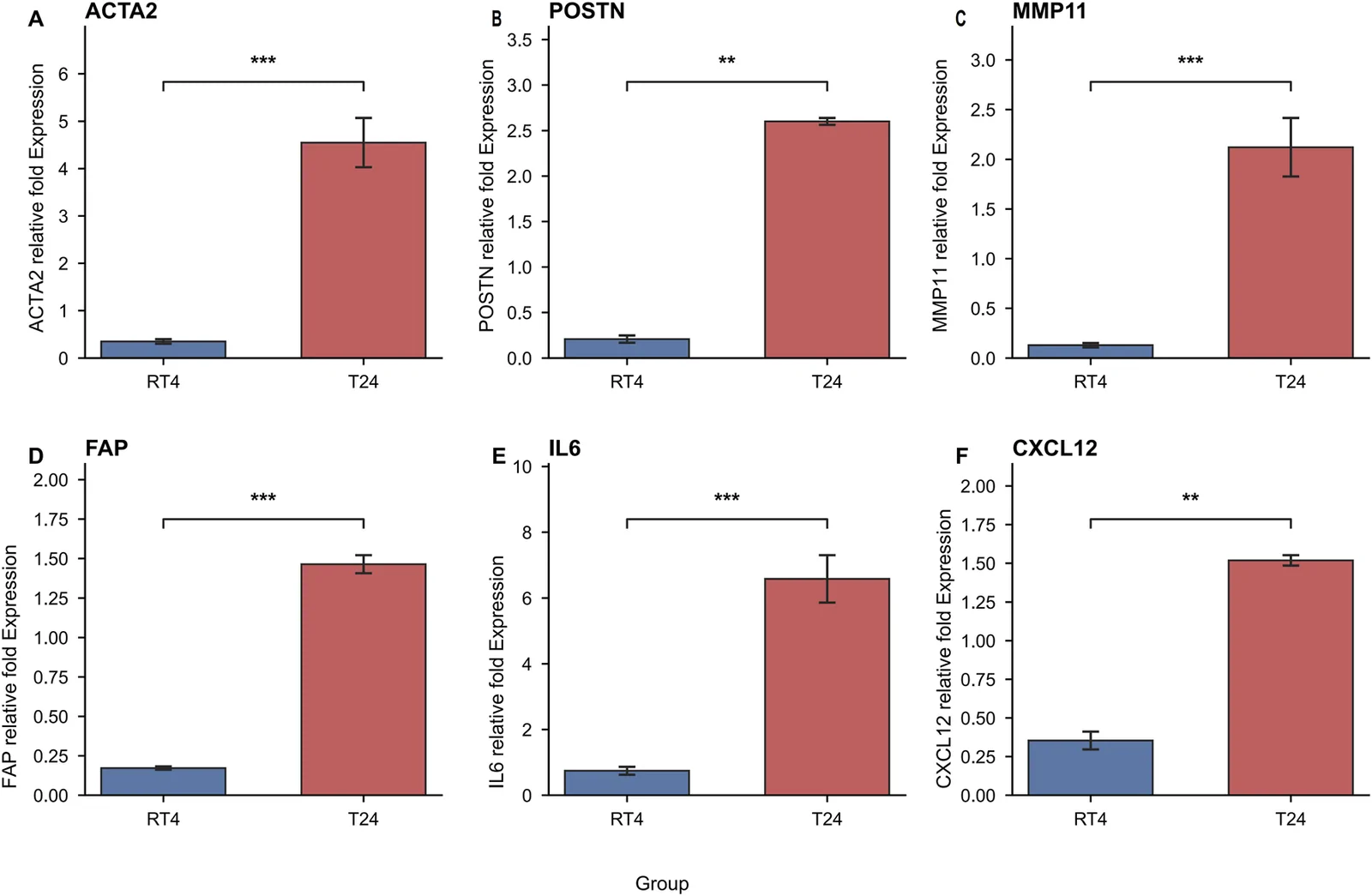
Relative expression of target genes in RT4 and T24 cells determined by RT–qPCR.Relative fold expression of ACTA2 **(A)** POSTN **(B)** MMP11 **(C)** FAP **(D)** IL6 **(E)**, and CXCL12 **(F)** was determined in RT4 and T24 cells. Expression was normalized to GAPDH using the 2-△Ct method and scaled by 103 for visualization. Bars represent the mean ± SD of three replicates per group. Statistical comparisons were performed on ΔCt values using unpaired two-sided Welch’s t-tests, followed by Benjamini–Hochberg correction for multiple comparisons. **P < 0.01; ***P < 0.001.

To obtain preliminary *in vitro* support for the transcriptomic analysis, we examined the expression of six CAF-related genes in RT4 and T24 bladder cancer cells by RT-qPCR (Figure 9). Compared with RT4 cells, T24 cells showed higher expression of several genes related to extracellular matrix remodeling and fibroblast-like activation. The relative expression levels of ACTA2, POSTN, MMP11, and FAP were increased respectively. The inflammatory and chemokine-related genes IL6 and CXCL12 were also elevated in T24 cells, compared to RT4 cells. These results were generally consistent with the bioinformatic findings, suggesting that aggressive bladder cancer cells may display stronger ECM-remodeling and inflammatory signaling features. This experiment provides supportive, preliminary evidence for the activation of myCAF- and iCAF-associated molecular programs in bladder cancer progression. It should be noted that elevated expression of ACTA2, FAP, and POSTN in T24 cells may partly reflect epithelial–mesenchymal transition (EMT) programs rather than canonical CAF identity; accordingly, this *in vitro* experiment represents cell-autonomous molecular evidence and does not substitute for validation in primary CAFs or CAF-conditioned fibroblasts.

## Discussion

This comprehensive single-cell transcriptomic investigation of the BLCA TME provides the most systematic molecular characterization to date of CAF heterogeneity and immune regulatory function in bladder cancer. Our principal findings are: (1) two transcriptionally distinct fibroblast states—Homeostatic and CCL2-high activated-like—are identified in adjacent-tissue BLCA specimens by exploratory single-cell analysis of a publicly available dataset (one specimen per condition); (2) a pre-specified six-gene myCAF-associated score tracks pathological stage in TCGA-BLCA but does not retain independent prognostic significance after age and stage adjustment; (3) this unadjusted stage association is not replicated in an independent bulk cohort (GSE31684); (4) exploratory LIANA ligand–receptor analysis identifies ECM, inflammatory, and protease-inhibitor fibroblast-outgoing interactions; and (5) RT–qPCR in contrasting bladder cancer cell lines provides preliminary cell-autonomous evidence for differential expression of myCAF- and CCL2-high-associated gene programs, with the caveat that some signal may reflect EMT.

The two adjacent-tissue fibroblast states identified here—Homeostatic (COL1A1/DCN-enriched) and CCL2-high activated-like—map onto a well-established spectrum in the CAF heterogeneity literature. The homeostatic state shares features with quiescent or “normal-like” fibroblasts described in pancreatic (Öhlund et al., 2017) and breast cancers (Costa et al., 2018), characterised by high ECM-structural gene expression and low inflammatory activation. The CCL2-high activated-like state, defined by elevated CCL2 (log2FC = 0.806, FDR = 8.08 × 10^−11^), is consistent with an inflammatory activation program analogous to published iCAF signatures enriched in cytokine and chemokine genes (Öhlund et al., 2017). Importantly, however, neither a myofibroblastic (myCAF) nor an antigen-presenting (apCAF) state was resolved in the available single-cell data, which comprised one adjacent-tissue and one tumor specimen with no fibroblast-like cells retained in the tumor specimen. This sampling constraint limits the interpretive scope of the current findings. Whether richer multi-specimen datasets would resolve additional fibroblast states analogous to those described in other cancer types (Costa et al., 2018; Elyada et al., 2019) remains an open question. Systematic molecular profiling of transcriptionally distinct subpopulations has been applied as a biomarker discovery strategy across diverse inflammatory disease contexts, supporting the translational value of rigorous cellular gene-expression characterisation (Wang et al., 2025).

The six-gene myCAF-associated score (ACTA2, COL1A1, MMP11, MYL9, TAGLN, TPM2) consistently tracked pathological stage across TCGA-BLCA and GSE13507, aligning with the well-established association between ECM-remodelling stromal programs and advanced disease stage (Mariathasan et al., 2018; Robertson et al., 2017). TGF-β signalling is a central mediator of fibroblast activation in the BLCA TME (Mariathasan et al., 2018), and the six score genes are functionally consistent with TGF-β-driven myofibroblastic programs described in the CAF literature (Öhlund et al., 2017; Biffi et al., 2019). However, the score did not retain independent prognostic significance after age and stage adjustment (adjusted HR = 1.03, P = 0.748) and was not associated with overall survival in an independent cohort (GSE31684; log-rank P = 0.443). These findings indicate that the score captures stage-related stromal biology rather than providing independent prognostic information, and underscore the caution required when interpreting unadjusted survival associations derived from publicly available datasets without prospective validation.

CXCL12–CXCR4 signalling represents a published mechanism by which CAF-derived chemokines can retain CD8^+^ T cells in the stromal compartment and prevent tumour-nest infiltration (Zhang et al., 2023; Feig et al., 2013), consistent with the immune-excluded phenotype described in BLCA. The CCL2-high activated-like fibroblast state identified here showed elevated CCL2 expression; whether these cells also upregulate CXCL12 cannot be confirmed from the present single-cell data alone, as CXCL12 was not a defining marker of either retained state. CXCR4 antagonists (plerixafor/AMD3100) have demonstrated pre-clinical efficacy in reversing T cell exclusion in other tumour models (Biffi et al., 2019; Dominguez et al., 2020), and further characterisation of the CXCL12-producing stromal compartment in BLCA may inform combinatorial ICI strategies. Based on ligand–receptor interaction scores computationally inferred from scRNA-seq data by LIANA, the CXCL12–CXCR4 axis is a candidate interaction; this represents a modelled prediction requiring empirical spatial and functional validation.

Diffusion pseudotime analysis positioned the CCL2-high activated-like state toward the later end of a within-sample state continuum (median DPT 0.813 vs. 0.672 for homeostatic fibroblasts), with FBLN1 as the strongest negatively correlated dynamic marker (rho = −0.808) (Haghverdi et al., 2016). This ordering is consistent with a transition from a quiescent ECM-maintaining programme toward greater inflammatory activation, paralleling trajectories described in published CAF studies (Öhlund et al., 2017; Costa et al., 2018); analogous transcriptomic identification of disease-relevant gene programmes has been applied across diverse experimental models and organ systems, underscoring the broad utility—and context-specific interpretive limits—of such approaches (Chen et al., 2025). It must be emphasised that rooted diffusion pseudotime applied to a single specimen is a within-sample state-ordering tool and does not establish a temporal lineage, a conversion event, or a causal relationship between states. Prospective studies with paired longitudinal sampling and lineage-tracing approaches would be required to characterise the true developmental dynamics of these fibroblast states in BLCA.

Limitations of this study include: the use of frozen dissociated tissue for scRNA-seq, which may incompletely capture highly matrix-embedded CAF populations; the absence of spatial transcriptomic data to validate the CXCL12 gradient model *in situ*; the cross-sectional study design precluding longitudinal assessment of CAF state evolution during therapy; and the need for functional validation of the identified CAF-immune regulatory mechanisms through *in vitro* co-culture and *in vivo* mouse model experiments. Future studies integrating 10x Visium spatial transcriptomics, primary CAF isolation from multi-stage BLCA specimens, and paired pre-/post-ICI therapy scRNA-seq will be essential to resolve additional fibroblast states and characterise their causal contributions to BLCA immunotherapy resistance.

## Conclusion

This exploratory single-cell transcriptomic analysis of publicly available BLCA data identifies two transcriptionally distinct fibroblast states in adjacent-tissue specimens and characterises their marker profiles, within-sample pseudotime relationships, and ligand–receptor interaction patterns. A pre-specified six-gene myCAF-associated score tracks pathological stage across multiple public cohorts but does not retain independent prognostic significance after covariate adjustment, and was not replicated for overall survival in an independent validation cohort. RT–qPCR in contrasting bladder cancer cell lines provides preliminary cell-autonomous molecular evidence consistent with the bioinformatic findings, while acknowledging that elevated expression of myCAF-associated markers in cancer cells may partly reflect EMT programs. Together, these findings constitute an exploratory computational and molecular framework; prospective studies incorporating primary CAF isolation, spatial transcriptomics, and adequately powered clinical cohorts are required before therapeutic implications can be drawn.

## Data Availability

The datasets presented in this study can be found in online repositories. The names of the repository/repositories and accession number(s) can be found in the article/Supplementary Material.

## References

[B1] Alfred W. J. Lebret T. Compérat E. M. Cowan N. C. De Santis M. Bruins H. M. (2017). Updated 2016 EAU guidelines on muscle-invasive and metastatic bladder cancer. Eur. Urol. 71 (3), 462–475. 10.1016/j.eururo.2016.06.020 27375033

[B2] Babjuk M. Burger M. Capoun O. Cohen D. Compérat E. M. Dominguez Escrig J. L. (2022). European association of urology guidelines on non-muscle-invasive bladder cancer. Eur. Urol. 81 (1), 75–94. 10.1016/j.eururo.2021.08.010 34511303

[B3] Barrett T. Wilhite S. E. Ledoux P. Evangelista C. Kim I. F. Tomashevsky M. (2013). NCBI GEO: archive for functional genomics data Sets—Update. Nucleic Acids Res. 41 (D1), D991–D995. 10.1093/nar/gks1193 23193258 PMC3531084

[B4] Biffi G. Oni T. E. Spielman B. Hao Y. Elyada E. Park Y. (2019). IL1-Induced JAK/STAT signaling is antagonized by TGFβ to shape CAF heterogeneity in pancreatic ductal adenocarcinoma. Cancer Discov. 9 (2), 282–301. 10.1158/2159-8290.CD-18-0710 30366930 PMC6368881

[B5] Cao J. Spielmann M. Qiu X. Huang X. Ibrahim D. M. Hill A. J. (2019). The single-cell transcriptional landscape of Mammalian organogenesis. Nature 566 (7745), 496–502. 10.1038/s41586-019-0969-x 30787437 PMC6434952

[B6] Chen Z. Zhou L. Liu L. Hou Y. Xiong M. Yang Y. (2020). Single-cell RNA sequencing highlights the role of inflammatory cancer-associated fibroblasts in bladder urothelial carcinoma. Nat. Commun. 11 (1), 5077. 10.1038/s41467-020-18916-5 33033240 PMC7545162

[B7] Chen J. Li Z. Liu X. Hu T. Gao N. Zhang W. (2025). Potential common key genes associated with myocardial dysfunction and brain injury following cardiac arrest resuscitation in a rat model. World J. Emerg. Med. 16 (3), 231–238. 10.5847/wjem.j.1920-8642.2025.055 40406296 PMC12093431

[B8] Costa A. Kieffer Y. Scholer-Dahirel A. Pelon F. Bourachot B. Cardon M. (2018). Fibroblast heterogeneity and immunosuppressive environment in human breast cancer. Cancer Cell 33 (3), 463–479.e11. 10.1016/j.ccell.2018.01.011 29455927

[B9] Dimitrov D. Türei D. Garrido-Rodriguez M. Burmedi P. L. Nagai J. S. Boys C. (2022). Comparison of methods and resources for cell-cell communication inference from single-cell RNA-seq data. Nat. Commun. 13 (1), 3224. 10.1038/s41467-022-30755-0 35680885 PMC9184522

[B10] Dominguez C. X. Müller S. Keerthivasan S. Koeppen H. Hung J. Gierke S. (2020). Single-cell RNA sequencing reveals stromal evolution into LRRC15+ myofibroblasts as a determinant of patient response to cancer immunotherapy. Cancer Discov. 10 (2), 232–253. 10.1158/2159-8290.CD-19-0644 31699795

[B11] Elyada E. Bolisetty M. Laise P. Flynn W. F. Courtois E. T. Burkhart R. A. (2019). Cross-species single-cell analysis of pancreatic ductal adenocarcinoma reveals antigen-presenting cancer-associated fibroblasts. Cancer Discov. 9 (8), 1102–1123. 10.1158/2159-8290.CD-19-0094 31197017 PMC6727976

[B12] Feig C. Jones J. O. Kraman M. Wells R. J. B. Deonarine A. Chan D. S. (2013). Targeting CXCL12 from FAP-Expressing carcinoma-associated fibroblasts synergizes with anti-PD-L1 treatment in pancreatic cancer. Proc. Natl. Acad. Sci. U. S. A. 110 (50), 20212–20217. 10.1073/pnas.1320318110 24277834 PMC3864274

[B13] Haghverdi L. Büttner M. Wolf F. A. Buettner F. Theis F. J. (2016). Diffusion pseudotime robustly reconstructs lineage branching. Nat. Methods 13 (10), 845–848. 10.1038/nmeth.3971 27571553

[B14] Hao Y. Hao S. Andersen-Nissen E. Mauck W. M. 3rd Zheng S. Butler A. (2021). Integrated analysis of multimodal single-cell data. Cell 184 (13), 3573–3587.e29. 10.1016/j.cell.2021.04.048 34062119 PMC8238499

[B15] Kalluri R. (2016). The biology and function of fibroblasts in cancer. Nat. Rev. Cancer 16 (9), 582–598. 10.1038/nrc.2016.73 27550820

[B16] Li Q. Qu Y. Xue J. Kang H. Lyu C. (2025). Exploring lipid-modifying therapies for sepsis through the modulation of circulating inflammatory cytokines: a Mendelian randomization study. World J. Emerg. Med. 16 (3), 256–261. 10.5847/wjem.j.1920-8642.2025.045 40406288 PMC12093423

[B17] Love M. I. Huber W. Anders S. (2014). Moderated estimation of fold change and dispersion for RNA-Seq data with DESeq2. Genome Biol. 15 (12), 550. 10.1186/s13059-014-0550-8 25516281 PMC4302049

[B18] Mariathasan S. Turley S. J. Nickles D. Castiglioni A. Yuen K. Wang Y. (2018). TGFβ attenuates tumour response to PD-L1 blockade by contributing to exclusion of T cells. Nature 554 (7693), 544–548. 10.1038/nature25501 29443960 PMC6028240

[B19] McInnes L. Healy J. Melville J. (2018). UMAP: uniform manifold approximation and projection for dimension reduction. J. Open Source Softw. 3 (29), 861. 10.21105/joss.00861

[B20] Öhlund D. Handly-Santana A. Biffi G. Elyada E. Almeida A. S. Ponz-Sarvise M. (2017). Distinct populations of inflammatory fibroblasts and myofibroblasts in pancreatic cancer. J. Exp. Med. 214 (3), 579–596. 10.1084/jem.20162024 28232471 PMC5339682

[B21] Powles T. Park S. H. Voog E. Caserta C. Valderrama B. P. Gurney H. (2020). Avelumab maintenance therapy for advanced or metastatic urothelial carcinoma. N. Engl. J. Med. 383 (13), 1218–1230. 10.1056/NEJMoa2002788 32945632

[B22] Robertson A. G. Kim J. Al-Ahmadie H. Bellmunt J. Guo G. Cherniack A. D. (2017). Comprehensive molecular characterization of muscle-invasive bladder cancer. Cell 171 (3), 540–556.e25. 10.1016/j.cell.2017.09.007 28988769 PMC5687509

[B23] Rosenberg J. E. Hoffman-Censits J. Powles T. van der Heijden M. S. Balar A. V. Necchi A. (2016). Atezolizumab in patients with locally advanced and metastatic urothelial carcinoma who have progressed following treatment with platinum-based chemotherapy: a single-arm, multicentre, phase 2 trial. Lancet 387 (10031), 1909–1920. 10.1016/S0140-6736(16)00561-4 26952546 PMC5480242

[B24] Subramanian A. Tamayo P. Mootha V. K. Mukherjee S. Ebert B. L. Gillette M. A. (2005). Gene set enrichment analysis: a knowledge-based approach for interpreting genome-wide expression profiles. Proc. Natl. Acad. Sci. U. S. A. 102 (43), 15545–15550. 10.1073/pnas.0506580102 16199517 PMC1239896

[B25] Sung H. Ferlay J. Siegel R. L. Laversanne M. Soerjomataram I. Jemal A. (2021). Global cancer statistics 2020: GLOBOCAN estimates of incidence and mortality worldwide for 36 cancers in 185 countries. CA Cancer J. Clin. 71 (3), 209–249. 10.3322/caac.21660 33538338

[B26] Traag V. A. Waltman L. van Eck N. J. (2019). From louvain to leiden: guaranteeing well-connected communities. Sci. Rep. 9 (1), 5233. 10.1038/s41598-019-41695-z 30914743 PMC6435756

[B27] Wang C. Yang D. Zhu Y. Yang Q. Liu T. Liu X. (2025). Circulating circular RNAs act as potential novel biomarkers for sepsis secondary to pneumonia: a prospective cohort study. World J. Emerg. Med. 16 (2), 144–152. 10.5847/wjem.j.1920-8642.2025.033 40135204 PMC11930550

[B28] Wolf F. A. Angerer P. Theis F. J. (2018). SCANPY: large-scale single-cell gene expression data analysis. Genome Biol. 19 (1), 15. 10.1186/s13059-017-1382-0 29409532 PMC5802054

[B29] Yu G. Wang L. G. Han Y. He Q. Y. (2012). clusterProfiler: an R package for comparing biological themes among gene clusters. OMICS 16 (5), 284–287. 10.1089/omi.2011.0118 22455463 PMC3339379

[B30] Zhang Z. Yu Y. Zhang Z. Li D. Liang Z. Wang L. (2023). Cancer-associated fibroblasts-derived CXCL12 enhances immune escape of bladder cancer through inhibiting P62-mediated autophagic degradation of PDL1. J. Exp. Clin. Cancer Res. 42 (1), 316. 10.1186/s13046-023-02900-0 38001512 PMC10675892

[B31] Zheng C. Zheng L. Yoo J. K. Guo H. Zhang Y. Guo X. (2017). Landscape of infiltrating T cells in liver cancer revealed by single-cell sequencing. Cell 169 (7), 1342–1356.e16. 10.1016/j.cell.2017.05.035 28622514

