# Supplementary material for: Single-cell transcriptomics deciphers cancer-associated fibroblast heterogeneity and immune regulatory mechanisms in the bladder cancer tumor microenvironment
Source: Front Cell Dev Biol. 2026 Aug 28;14:1889050. doi: 10.3389/fcell.2026.1889050 (PMC13562231; doi:10.3389/fcell.2026.1889050)
Supplement: Supplementary file 1 [file Table1.docx]

Supplementary Table 1

|  | **Forward primer 5'-3'** | **Reverse primer 5'-3'** |
| --- | --- | --- |
| **ACTA2** | CCCAGACATCAGGGAGTAATGG | TCTATCGGATACTTCAGCGTCA |
| **POSTN** | TGCCCAGCAGTTTTGCCCAT | CGTTGCTCTCCAAACCTCTA |
| **MMP11** | CCGCAACCGACAGAAGAGG | ATCGCTCCATACCTTTAGGGC |
| **FAP** | CCAGCAATGATCGGCAATTTGT | GTTCTGGTCAAGTTCATCTGTG |
| **IL6** | ACTCACCTCTTCAGAACGAATTG | CCATCTTTGGAAGGTTCAGGTTG |
| **CXCL12** | ATTCTCAACACTCCAAACTGTGC | ACTTTAGCTTCGGGTCAATGC |
| **GAPDH** | GGAGCGAGATCCCTCCAAAAT | GGCTGTTGTCATACTTCTCATGG |
